# Tracking Change: A Look at the Ecological Footprint of Antibiotics and Antimicrobial Resistance

**DOI:** 10.3390/antibiotics2020191

**Published:** 2013-03-27

**Authors:** Patricia L. Keen, David M. Patrick

**Affiliations:** 1 Department of Civil Engineering, University of British Columbia, 2002-6250 Applied Science Lane, Vancouver, BC V6T 1Z4, Canada; 2 School of Population & Public Health, University of British Columbia, 2206 East Mall Vancouver, BC V6T 1Z3, Canada; E-Mail: David.Patrick@bccdc.ca

**Keywords:** antibiotics, environment, antimicrobial resistance, health

## Abstract

Among the class of pollutants considered as ‘emerging contaminants’, antibiotic compounds including drugs used in medical therapy, biocides and disinfectants merit special consideration because their bioactivity in the environment is the result of their functional design. Antibiotics can alter the structure and function of microbial communities in the receiving environment and facilitate the development and spread of resistance in critical species of bacteria including pathogens. Methanogenesis, nitrogen transformation and sulphate reduction are among the key ecosystem processes performed by bacteria in nature that can also be affected by the impacts of environmental contamination by antibiotics. Together, the effects of the development of resistance in bacteria involved in maintaining overall ecosystem health and the development of resistance in human, animal and fish pathogens, make serious contributions to the risks associated with environmental pollution by antibiotics. In this brief review, we discuss the multiple impacts on human and ecosystem health of environmental contamination by antibiotic compounds.

## 1. Introduction

For millennia, long before humans made footprints on the earth’s surface, bacteria have been performing crucial biological services in diverse ecological niches. Humankind learned of the advantageous use of many natural processes in which bacteria play a pivotal role and forged a relationship that has served human enterprise ever since. However, the consequences of this relationship are not exclusively positive. A comparatively small proportion of bacterial species are pathogens but some of these are responsible for infectious diseases that can have devastating impacts on human and animal health. Humans have exploited the activities of antibiotic compounds, many of which are derived from nature, to fight bacterial infections for centuries. Despite human efforts to manipulate nature, our unintended consequences on ecological processes in the natural environment can influence human health outcomes. Development of antibiotic resistance in pathogens is a shining example of health issues in which understanding the central role of the environment is crucial to managing this potential risk. Here we discuss recent contributions to our understanding of relationships between health, antibiotic resistance and the environment.

Although widespread use of antibiotic drugs to treat human infections grew tremendously since penicillin was introduced into medical therapy [1], the beneficial antibiotic properties of many bacteria and plant species have been known for a very long time. Recent advances in analytical chemistry have provided evidence that tetracycline derived from *Streptomyces* bacteria in beer fermentation was used for medical treatment of infection in ancient (350–550 AD) Nubian patients [2]. The ancient Greeks and Romans valued wild indigo root as both a blue dye and for its ability to treat respiratory tract infections and some soft tissue infections [3]. Interestingly, woad obtained from the plant *Isatis tinctoria* produces blue dye similar to indigo and has antibiotic properties [4] for which historical documents confirm its use in traditional Chinese medicine and in European medicine to treat certain infections since the 14th century. *Chromobacterium violaceum* is a Gram-negative, heterotrophic bacterium common in soil or aquatic ecosystems in many tropical regions and frequently found in black water in the Amazon River [5]. This particular bacterial species is well-known for its purple pigment and has demonstrated antimicrobial properties that have activity against *Leishmania* species, *Mycobacterium tuberculosis* and some viruses [6]. Although many plant species are known to produce compounds with antibiotic activity, few show promise for applications in human medicine as potent broad-spectrum antimicrobials [7]. 

Processes that lead to the development of antibiotic resistance have likely occurred throughout all of microbial evolutionary history. Microbial analyses of rock surfaces of the Lechuguilla cave system in New Mexico, dating back some 4 million years, has revealed the presence of bacteria that are resistant to many structurally and chemically diverse antibiotics currently used in human medicine [8]. Phylogenetic analyses showed that the OXA genes which encode a class of β-lactamases that confers resistance to a broad range of β-lactam antibiotics have existed on plasmids for millions of years [9]. This evidence counters the popular argument that mobilization of antibiotic resistance genes is completely due to modern use of antibiotics in medical therapy. However, despite evidence that the evolutionary processes responsible for development of antibiotic resistance in environmental bacteria probably occurred for billions of years, it is clear that selection pressure has become more intense with increasing use and disposal of antibiotics and that this selection is geared more towards ensuring survival in hostile environments rather than improving fitness in slowly evolving populations [10,11,12]. Development of antibiotic resistance in bacteria is yet another example for which the metaphor of the ‘ecological footprint’ [13] aptly describes the increase in impacts on natural processes that occur as a result of human activities—in this case via underuse, overuse and misuse of antibiotics in medical therapy.

## 2. Why the Environment Matters

Ever-changing environmental conditions influence the biological, chemical, physical and ecological processes that govern the health of all ecosystem species. Just as humans must adapt to environmental stressors, the survival of healthy populations of bacteria depends on their ability to respond quickly to overcome environmental threats. Gene exchange is a common property of all bacteria. When human activities amplify the effects of exposure to stressors, the development of resistance to these represents one of the most striking illustrations of Darwinian selection and survival. Scale is an important factor in considering the response of organisms to environmental change. The effects of processes that occur at the cellular level often translate to effects at different environmental scales. For example, the mechanisms of development of antibiotic resistance in bacteria resident in biofilms lining the catheter tubes of a human patient can be very similar to those in the biofilms lining a wastewater treatment reactor. Microbes interact with a myriad of small molecules in their lifetime and it is likely that resistance develops as a self-defense mechanism, an altered response to chemical signals or as a way to metabolize molecules as a food source.

It is now accepted that resistance is a natural property of all bacteria [14,15,16] and the term ‘resistome’ is used to describe the framework that encompasses all forms of resistance and precursor elements [17]. More and more evidence is being collected to support the idea that the environment acts both as a reservoir for antibiotic resistance and a means by which this resistance can be broadly disseminated. Genes can move quickly through a bacterial population via vertical or horizontal transfer mechanisms and combined with the grand magnitude of the resistome, it is no wonder that bacteria can quickly adapt to resist new drugs soon after they are introduced for medical or agricultural use. Resistant bacteria, antibiotic resistance genes, degradative enzymes that work to inactivate antibiotics, and antibiotic molecules are present in the environment at all times and thus distinguishing naturally occurring resistance in organisms from resistance as a result of environmental pollution is a complicated task. Each of these contributors adds a layer of complexity to our understanding of the environmental footprint of antibiotics. 

## 3. Resistant Pathogens as Environmental Contaminants

Superbugs exist everywhere in nature. Several well-known pathogens have demonstrated disturbing trends for developing resistance to antimicrobials. Since Snow’s first epidemiological study of cholera in 1854 [18], water contamination with toxigenic *Vibrio cholerae* has been a well-understood risk to public health of ongoing relevance in many developing countries. *Vibrio cholerae* exists in the environment and, during its quiescent stage, distribution of the pathogen through bodies of water spreads the threat to human health, particularly when strains are resistant to multiple classes of antibiotics. Outbreaks of cholera are wide-spread in the developing world (particularly after natural disasters) and among the recent examples of emergence of multi-drug resistant strains of the pathogen are reports from India [19], China [20], and Haiti [21]. Although comparatively few of the species of bacteria in the environment are pathogens, those that have earned the reputation as ‘superbugs’ make substantial contributions to the environmental footprint of antibiotics by increasing the amount and diversity of drugs needed to combat infection, increasing the cost of treating illness and altering the ecological conditions of healthy functioning ecosystems.

Emergence of multi-drug resistance in community acquired pathogens such as *Mycobacterium tuberculosis* and *Streptococcus pneumoniae* illustrates a worrisome scenario in which the effectiveness of the antibiotics that previously successfully treated the infections has deteriorated over time. Nocosomial pathogens associated with hospital acquired infections, such as methicillin-resistant *Staphylococcus aureus*, *Pseudomonas aeruginosa* and Vancomycin-resistant *Enterococcus faecium* occur with high frequency of drug-resistance traits and have been detected in wastewater treatment plants that receive hospital effluent discharge [22,23,24,25]. The discharge of effluents from wastewater treatment plants provides the pathway for introduction of contaminants into the receiving environment. Given that secondary treatment of municipal wastewater depends on maintaining conditions for healthy populations of useful bacteria in reactors to degrade sewage, it is highly likely that some resistant pathogens could also be among the organisms released after waste processing.

Environment has multiple meanings. Ecosystems describe communities of living organisms and their relationship to the non-living environmental condition in which they reside. Hospitals and each of their sub-units represent unique ecosystems that, in some cases, have environmental conditions that facilitate rapid development of antibiotic resistance in pathogens. Among the hospital acquired pathogens, the emergence and spread of resistant strains of *Clostridium difficile* is demonstrating a disturbing upward trend. Increased multi-drug resistance and virulence in *Clostridium difficile* is well-documented [26,27,28] and this is of concern since outbreaks of *C. difficile* infections occur in long-term care facilities with increased frequency [29,30]. In addition, zoonotic transfer of pathogens via companion animals may be an under-recognized mode of transmission of organisms including *Clostridium difficile*. Although a common pathogen among several animal species, within the past decade, *C. difficile* has become accepted as an enteric pathogen in horses [31,32,33]. Humans and pets often share the same environment in close proximity making transmission of bacteria likely but particularly problematic if the same species of enteric pathogen can thrive in human and animal hosts. When infections that were previously associated with the hospital environment occur in the community, the implications connected with development of resistance in the pathogens responsible for these outbreaks amplify the public health concern. 

Another important pathogen that has a long history of association with hospital acquired infections is *Acinetobacter baumaii.* This species of gram negative bacilli are common soil dwelling organisms that are widely distributed in nature. Because they can survive under a broad range of environmental conditions and can exist for long periods of time on dry surfaces, the risk of transmission between individuals is high. Multi-drug resistant *Acinetobacter baumaii* is a rapidly emerging pathogen in health care settings where antimicrobial resistance has seriously limited options for treatment [34]. As a last resort for multi-drug infections, carbapenems are used to treat *Acinetobacter baumaii* associated infections however, carbapenem-resistant strains of the pathogen are being reported at an alarmingly high rate [35,36,37,38]. Outbreaks of severe multi-drug resistant *A. baumannii* were observed in military personnel active in the conflicts in Iraq and Afghanistan [39,40,41,42,43]. Originally, environmental exposures to soil-dwelling strains of the pathogen were thought to be responsible for the outbreaks. However, subsequent culture-based studies suggested that exposure in health care facilities is the more likely pathway of transmission between patients [44,45,46,47,48]. Environmental contamination plays a critical role as an important reservoir in outbreaks of *A. baumannii* and in extending the need for more frequent use and higher potency antibiotic drugs.

Microbes travel. Environmental transport of antibiotic resistant bacteria, especially human and animal pathogens, extends opportunities for exposure of non-target organisms in air, water, soil and sediment. People travel. Increased global mobility of humans and animals has influenced the rates of exposure to pathogens in unprecedented ways. 

## 4. Antimicrobial Resistance Genes as Environmental Contaminants

Environmental contamination by antibiotic compounds is inextricably linked to development of antimicrobial resistance in non-target species of bacteria. Whether the bacteria perform critical ecosystem services, pose a health threat as pathogens or have incompletely understood functions in nature, development of antimicrobial resistance as a result of human activities is problematic. Susceptibility characteristics of microbes can be altered by incorporation of genetic information encoding for resistance or by mutation in their DNA. Antibiotic resistance genes are recognized as important environmental contaminants [49,50,51]. Bacteria strains containing genes that code for resistance to fluoroquinolones [52,53,54,55,56], macrolides [57,58,59], sulfonamides [60,61,62] and trimethoprim [63,64] have been isolated from environmental samples. Resistance can spread as a result of distribution of resistant bacterial strains or genetic elements of resistance throughout the receiving environment, evolution and selection of new resistant strains or the amplification of pre-existing resistant strains of bacteria. Introduction of antibiotics as environmental contaminants have important influences on these processes that alter abundance of resistance genes in the environment in multiple ways. 

Genes of some strains of bacteria encode for the production of enzymes that are responsible for resistance to antibiotics which have the four-carbon beta lactam ring in their chemical structure. These enzymes, known as beta lactamases, are capable of breaking the ring structure that is common to antibiotics, such as penicillins, cephamycins and carbapenems, thereby inactivating the antimicrobial properties of the molecule. Extended-spectrum beta lactamases (ESBLs) are a sub-group of this class of enzymes that are often plasmid encoded and also carry genetic traits for resistance to cephalosporins and other antibiotic drugs used for treatment of serious pathogenic infections of human patients. These classes of antibiotics represent the last resort in treatment for some infections. Incidence of infection by ESBL-producing organism is being reported with increasing frequency [65,66,67]. This is an extremely important concern in the medical community since the range of antibiotic treatment options is progressively shrinking. One of the latest examples of this disturbing trend is the emergence of New Delhi metallo-beta-lactamase (NDM-1) in some strains of Gram negative bacteria that have demonstrated resistance to virtually all antibiotic drugs in common use. The role of the environment in the transport and dissemination of bacteria producing deactivating enzymes cannot be overlooked given that ESBL-producing *Escherichia coli* has already been isolated in samples collected from wastewater treatment effluents [68] and in isolates collected from household pets [69].

## 5. Antibiotic Drugs as Environmental Contaminants

It is accepted knowledge that antibiotics are present as contaminants in a variety of environmental systems [70,71,72,73,74,75,76,77]. Among the class of pollutants referred to as ‘emerging contaminants’, antibiotic compounds have a suspicious reputation because their biological activity is an intrinsic characteristic of their functional design. Antibiotics are introduced into the environment via multiple pathways that include effluents from disposal of human waste, waste from agricultural food animal production and aquaculture of finfish, direct application to some plants, industrial effluents from pharmaceutical production, agricultural run-off and disposal of ethanol production waste products. Acute and chronic bioassays have provided evidence that environmental exposure to low concentrations of some antibiotic drugs have toxic effects in species such as *Daphnia magna* [78], *Selenastrum capricornutum* [79] and *Artemia* [80]. Toxicity evaluations of enrofloxacin and ciprofloxacin using representatives of four photoautotrophic aquatic species found that enrofloxacin did not inhibit growth although toxic responses in some of the macrophytes were observed for exposure to ciprofloxacin at the same environmentally relevant concentrations [81]. 

Failure to detect antibiotics in environmental samples (especially those with high organic content) does not mean antibiotic residues are not present. The diverse chemical and structural properties of antibiotic compounds in environmental sample matrices make chemical determination problematic in many circumstances. Biodegradation [82,83,84], photodegradation [85,86,87,88,89], chemical complexation or chelation [90,91,92,93,94] and adsorption to particulate matter [95,96,97,98,99,100,101,102,103,104,105] alter the concentrations of antibiotic residues that can be reliably measured in samples of environmental origin. The current analytical method of choice is liquid chromatography combined with tandem mass spectrometry using a variety of extraction methods, frequently solid phase extraction techniques. Despite excellent improvements in analytical determination of antibiotic residues in environmental samples, analyte recoveries, detection limits and reproducibility can be highly variable.

The discharge of effluents from wastewater treatment plants represents important point sources of contaminants in the environment. Wastewater treatment plants have been described as ‘hotspots’ for antibiotics [106] and for antimicrobial resistance [107] although some treatment options appear promising for reducing the load of antibiotic residues that could be delivered to the receiving environment. Processes for the removal of oxytetracycline [108], sulfamethoxazole [109,110], trimethoprim [111], and some fluoroquinolones [112,113] have been demonstrated. Biological waste treatment processes rely on complex ecological interactions among the microbial species present in the system reactors. Our understanding of these ecological factors is growing and processes to treat wastewater have been shown to influence the contribution of antimicrobial resistance elements and resistant strains of bacteria released into the environment [114,115,116].

Direct application of antibiotic compounds can contribute to the contaminant load on the receiving environment. Oxytetracycline and streptomycin are frequently used in orchards to prevent Erwinia infection in apples and pears. Alternatives to the application of antibiotics on trees to control conditions such as fire blight include spraying orchards with a copper sulphate solution known as ‘Bordeaux mixture’. Although the practice is common and effective in the treatment of fire blight, recent studies have demonstrated that long-term application of copper to agricultural soil altered microbial community structure and altered gene expression in some soil macrofauna [117]. As in the case of human and veterinary medicine, treatment of infections in plants requires judicious use of antimicrobials such that the beneficial ecosystem services provided by a plethora of bacteria in the environment are not compromised.

The original concept behind the ecological footprint model was to compare human demands on nature with nature’s ability to regenerate the resources needed to accommodate human consumption for lifestyle needs. Under the rubric of mitigating anthropogenic impacts on climate change and reducing human dependence on limited resources of fossil fuels, production of ethanol is one case where effects to reduce human ecological footprint may contribute to the increase in the microbial ‘resistance footprint’. Conversion of corn starch to ethanol is highly susceptible to contamination by bacteria that will compete with yeast during the fermentation process [118]. In order to control excessive growth of bacterial populations in the production process, antibiotics such as penicillin, erythromycin, virginiamycin and tylosin are frequently added to the tanks where corn mash is mixed with warm water to ferment ethanol. The waste by-product of the ethanol fermentation is nutrient-rich corn mash known as ‘distillers grains’ and is a common ingredient in livestock or poultry feed. Evidence supports the hypothesis that macrolide antibiotics in dried distillers’ grains can persist through the fermentation process and remain active after incorporation in livestock feed [119,120,121]. 

## 6. Conclusion

Environmental contamination by antibiotics is but one factor in the equation that defines the health consequences of antibiotic resistance in bacteria. The combined impact of resistant strains of bacteria, antibiotic resistance genes, degradative enzymes that inactivate antibiotics, and antibiotic compounds can have profound influence on human and ecosystem health. It will take a concerted effort involving antibiotic stewardship, judicious use of antibiotics in human and animal medicine, surveillance of drug use and the incidence of antimicrobial resistance, public awareness campaigns, and government commitment to leading coordinated initiatives for society to be protected from the deleterious consequences of excessive development of antibiotic resistance in pathogens. The environmental footprint of antibiotics must be minimized to ensure that the drugs that we use in human and veterinary medicine remain effective. This is our path of “least resistance”. 
